# Malakoplakia in Immunocompromised Hosts: A Case Series and Literature Review

**DOI:** 10.3390/diseases14080284

**Published:** 2026-08-08

**Authors:** Ahmed Bishara, Huma Saeed, Layan Akkielah, Noor BuMurah, David K. Driman, Michael Silverman, Reza Rahimi Shahmirzadi

**Affiliations:** 1 Schulich School of Medicine & Dentistry, Western University, London, ON N6A 5C1, Canada; ahmad.bishara@live.com (A.B.); huma.saeed@lhsc.on.ca (H.S.); noor.bumurah@lhsc.on.ca (N.B.); david.driman@lhsc.on.ca (D.K.D.); michael.silverman@sjhc.london.on.ca (M.S.); 2 Division of Infectious Diseases, Western University, London, ON N6A 4V2, Canada; 3 Division of Infectious Diseases, Department of Internal Medicine, Imam Abdulrahman Bin Faisal University, Dammam 34212, Saudi Arabia; 4 Pathology and Laboratory Medicine, London Health Sciences Centre, London, ON N6A 5A5, Canada

**Keywords:** malakoplakia, immunocompromised host, inflammatory bowel disease, acute myeloid leukemia, solid organ transplantation

## Abstract

Background: Malakoplakia is a rare chronic granulomatous inflammatory disorder characterized by defective macrophage phagolysosomal activity and accumulation of Michaelis–Gutmann bodies on histopathology. It occurs predominantly in immunocompromised individuals and may mimic infectious, inflammatory, or neoplastic processes, creating significant diagnostic challenges. We describe four cases of malakoplakia occurring in distinct immunocompromised states and review the published literature to better characterize its clinical spectrum, management, and outcomes. Methods: We conducted a retrospective case series of four patients diagnosed with histologically confirmed malakoplakia at our institution. Cases occurred in the setting of liver transplantation, kidney transplantation, relapsed acute myeloid leukemia, and ulcerative colitis treated with immunosuppressive therapy. A literature review was performed using PubMed and Google Scholar to identify published cases of malakoplakia in immunocompromised hosts. Demographic, clinical, microbiological, therapeutic, and outcome data were extracted and analyzed descriptively. Results: Four patients with malakoplakia involving the gastrointestinal tract or renal allograft were identified. Clinical presentations ranged from incidental endoscopic findings and tumor-like colonic masses to recurrent bacteremia and graft dysfunction. Histopathologic examination demonstrated characteristic Michaelis–Gutmann bodies in all cases. Management included antimicrobial therapy, observation, and surgical intervention when necessary. Two patients achieved complete clinical and histologic resolution, one required transplant nephrectomy because of persistent allograft infection, and one died from progressive acute myeloid leukemia. Combined with 48 cases identified in the literature, 52 patients were analyzed. The gastrointestinal tract was the most frequently affected site (76.9%), followed by the genitourinary tract (19.2%). Malignancy (32.7%), solid-organ transplantation (26.9%), and autoimmune disease (21.2%) were the most common underlying conditions. Conclusions: Malakoplakia should be considered in the differential diagnosis of mass lesions, persistent infections, and inflammatory lesions in immunocompromised patients, particularly those with malignancy or receiving immunosuppressive therapy. Early histopathologic diagnosis is essential to distinguish malakoplakia from malignancy and guide appropriate management. Our findings highlight the heterogeneous clinical manifestations and outcomes of this uncommon condition and emphasize the importance of multidisciplinary evaluation in affected patients.

## 1. Introduction

The rare chronic granulomatous condition known as malakoplakia is caused by macrophages’ impaired phagolysosomal function, resulting in an inability to completely digest phagocytized bacteria. Malakoplakia has been reported in various organ systems, including the gastrointestinal tract, bone, lungs, and skin; it most commonly involves the gastrointestinal and genitourinary tracts [1,2,3]. It is associated with Gram-negative infections, mainly due to *Escherichia coli* (*E. coli*). However, malakoplakia lesions have also been found to harbor *Klebsiella pneumoniae*, *Mycobacterium tuberculosis, Proteus* spp., *Rhodococcus equi, Staphylococcus aureus*, and *Pseudomonas aeruginosa* [4,5]. Histologically, the lesions are identified by macrophages that have basophilic inclusion structures called Michaelis–Gutmann bodies, which are calcified lysosomes [3].

Malakoplakia may form tumor-like masses, resembling neoplasms, making diagnosis challenging. It is more common in immunocompromised patients and is more likely to occur in transplant recipients. Additionally, it may develop as a result of cancer, autoimmune diseases, HIV, or any other clinical condition that calls for steroid and therapeutic immunosuppression [6,7,8]. Management typically involves prolonged antimicrobial therapy, optimization or reduction in immunosuppressive medications when feasible, and surgical intervention in selected cases with localized, refractory, or advanced disease [9,10].

We report a case series highlighting the heterogeneous clinical manifestations of malakoplakia across a range of immunocompromised states, including solid-organ transplantation, hematologic malignancy, and chronic immunosuppressive therapy for autoimmune and inflammatory diseases. We present four patients with malakoplakia developing in different immunocompromised situations: liver transplantation, renal transplantation, acute myeloid leukemia, and ulcerative colitis with biological therapy. This illustrates the clinicopathological spectrum, diagnostic challenges, treatment, and prognosis and includes a review of the literature.

## 2. Literature Review

### 2.1. Materials and Methods

We performed a review of the literature and searched databases including PubMed and Google Scholar using keywords: “malakoplakia,” “colonic malakoplakia,” “renal malakoplakia,” “liver transplant,” “kidney transplant,” “immunosuppression,” “’acute myeloid leukemia”, and “hematologic malignancy.” The search included studies published from database inception using combinations of the following search terms: (‘malakoplakia’ OR ‘malacoplakia’) AND (‘immunocompromised’ OR ‘transplant’ OR ‘hematologic malignancy’ OR ‘acute myeloid leukemia’ OR ‘inflammatory bowel disease’ OR ‘ulcerative colitis’).” We included case reports, case series, and observational studies that reported confirmed malakoplakia by histopathological examination. Duplicate reports of previously documented cases and non-English publications without full-text access were not included. The literature was searched for reports of histopathologically confirmed malakoplakia in immunocompromised patients that included full-text articles. Only histopathologically confirmed cases of malakoplakia were included. Cases diagnosed solely on clinical or radiological findings without pathological confirmation were excluded. When reported, the presence of Michaelis–Gutmann bodies was considered confirmatory. Duplicate publications describing the same patient were identified by comparing demographic and clinical characteristics and were excluded. Studies with insufficient clinical information, duplicate reports, incomplete conference abstracts and non-English publications with no full text were not included. The evidence available was mostly in the form of case reports and small case series, which did not allow for formal quality assessment. From each case, we extracted a standardized dataset, including patient age, sex, underlying immunocompromising condition, affected organ system, clinical symptoms, infectious organisms (when available), treatment strategies, and clinical outcomes. We computed descriptive statistics for these variables.

Given the rarity of the condition and the predominance of case reports and small case series, a formal systematic review and risk-of-bias assessment were not performed.

### 2.2. Results

In addition to our four cases, we found 48 cases reported in the literature, leading to a total of 52 cases analyzed. The most frequent anatomic site was the gastrointestinal tract (N = 40, 76.9%), followed by the genitourinary tract (N = 10, 19.2%), maxilla (N = 1, 1.9%), and cutaneous tissue (N = 1, 1.9%). Mean age at time of diagnosis was 50.0 years; 26 (50.0%) of patients were male, and 26 (50.0%) were female. Malignancy, solid organ transplantation, autoimmune disease, immunosuppressive therapy, diabetes mellitus, infection, and HIV were the underlying clinical conditions in 17 cases (32.7%), 14 cases (26.9%), 11 cases (21.2%), nine cases (17.3%), seven cases (13.5%), four cases (7.7%), and one case (1.9%), respectively, of the total cohort. From the microbiological point of view, nine cases (17.3%) were reported specifically as culture-positive. Isolates of chronic inflammatory infectious organisms were *Escherichia coli*, *vancomycin-resistant Enterococcus (VRE)*, *Providencia* spp., *Candida albicans*, *Klebsiella pneumoniae*, and *Enterococcus faecium*. Of the total population, 16 patients (30.8% of the total population, or 40% of the institutional cases) were noted at the time of diagnosis to be undergoing active immunosuppressive therapy. The drugs reported as iatrogenic immunosuppressive agents and immunomodulators were anti-thymocyte globulin (ATG), methylprednisolone, basiliximab, mycophenolate mofetil, tacrolimus, prednisone, hydroxychloroquine, mycophenolate sodium, prednisolone, methotrexate, cyclophosphamide, and infliximab. As far as therapeutic management is considered, 20 patients (38.5%) were treated with antimicrobial therapy, including targeted antibiotics such as ciprofloxacin, cefepime, trimethoprim-sulfamethoxazole (TMP/SMX), tetracycline, minocycline, ceftriaxone, daptomycin, rifaximin, oral cefpodoxime, doxycycline and levofloxacin, while 10 (19.2%) received surgical management. Clinical outcomes were reported for 25 of the 52 patients. Among those with available follow-up data, malakoplakia resolved or improved in 24 patients, whereas one patient died due to progressive acute myeloid leukemia unrelated to malakoplakia. One patient in our case series died due to progressive relapsed acute myeloid leukemia and its associated complications rather than malakoplakia. Clinical outcomes and responses to treatment were not reported in the remaining cases (Table 1). The clinical characteristics, management strategies, and outcomes of the four patients in our case series are presented in (Table 2).

## 3. Case Presentations

### 3.1. Case Number One: Liver Transplant with Colonic Malakoplakia

A 56-year-old man with a history of primary biliary cholangitis underwent living donor liver transplantation in 2001, followed by orthotopic liver re-transplantation in 2004 for primary biliary cholangitis. His medical history was significant for type 2 diabetes mellitus, dyslipidemia, obstructive sleep apnea, iron deficiency anemia, and peptic ulcer disease. He remained on chronic immunosuppression with tacrolimus and mycophenolate mofetil.

**Figure 1:**
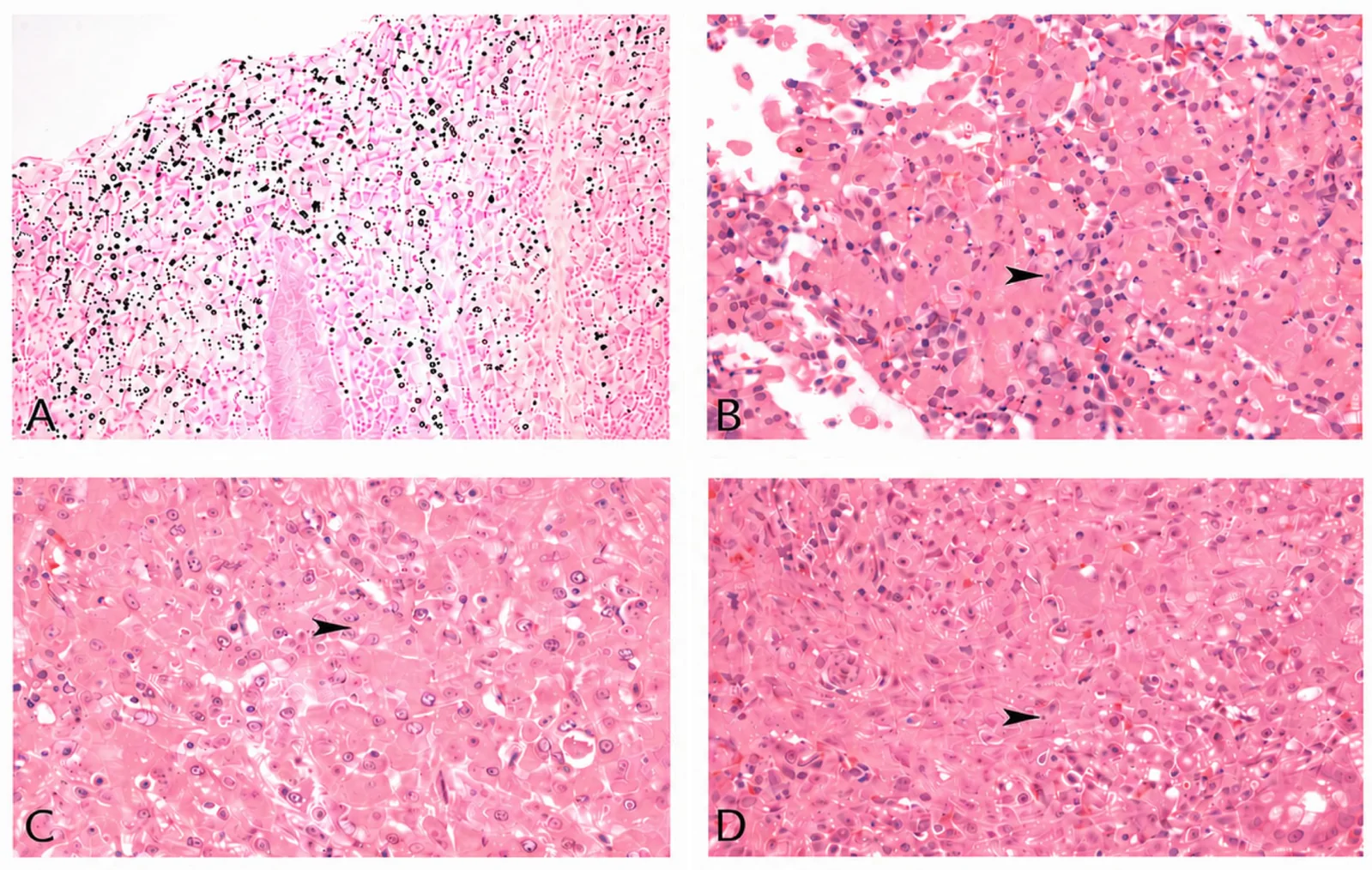
(**A**): Case number 1. Colonic mucosa, Von Kossa stain: Numerous Michaelis–Guttman bodies stain positively. (**B**): Case number 2. Colonic mucosa, H&E stain: Eosinophilic macrophages with the presence of Michaelis–Guttman bodies (arrowhead). (**C**): Case number 3. Kidney: Eosinophilic macrophages with the presence of Michaelis–Guttman bodies (arrowhead). (**D**): Case number 4. Colonic mucosa, H&E stain: Eosinophilic macrophages with the presence of Michaelis–Guttman bodies (arrowhead).

More than two decades after transplantation, the patient was admitted with acute upper gastrointestinal bleeding secondary to bleeding duodenal ulcers requiring endoscopic intervention and transfusion of three units of packed red blood cells. Helicobacter pylori testing was negative, and there was no significant NSAID use aside from low-dose aspirin, which was subsequently discontinued. Upper and lower gastrointestinal endoscopy pathologic examination of a rectal biopsy demonstrated extensive histiocytic infiltrates with Michaelis–Gutmann bodies (Sigma-Aldrich, St. Louis, MO, USA), consistent with malakoplakia (Figure 1A). He was not started on treatment for the malakoplakia.

After two months, the patient was transferred to another hospital to seek a second opinion, where laboratory investigations showed a white blood cell count of 10.2 × 10^9^/L, hemoglobin 144 g/L, platelet count 337 × 10^9^/L, creatinine 73 µmol/L, and tacrolimus level 4.8 ng/mL. Additional investigations excluded infectious etiologies, including mycobacterial infection, fungal infection, and Whipple disease.

Subsequent repeat colonoscopy with biopsies again showed malakoplakia; Ziehl–Neelsen and periodic acid–Schiff stains were negative. Tissue bacterial, fungal, and mycobacterial cultures were also negative, and QuantiFERON-TB Gold testing did not demonstrate evidence of tuberculosis infection. He was diagnosed with rectal malakoplakia in the setting of chronic post-transplant immunosuppression.

Although the patient remained clinically stable, persistent histopathological evidence of malakoplakia on repeat biopsy prompted initiation of antimicrobial therapy because TMP-SMX has favorable intracellular penetration and has been successfully used in previously reported cases. The patient was started on trimethoprim-sulfamethoxazole 160/800 mg tablet twice daily and tolerated therapy well without adverse effects. Following initiation of antimicrobial therapy, the patient reported significant improvement in gastrointestinal symptoms, and he completed a 3-month course of therapy without complications. Five months after completing treatment, follow-up colonoscopy and biopsies revealed no abnormalities, and at the last follow-up, the patient remained clinically asymptomatic.

### 3.2. Case Number Two: AML with Colonic Malakoplakia

A 70-year-old man with relapsed acute myeloid leukemia (AML) was admitted in late 2022 with hypercalcemia and acute kidney injury in the setting of relapsed acute myelomonocytic leukemia with leukemia cutis. He had initially been diagnosed with AML earlier that year and achieved remission following induction chemotherapy with daunorubicin and cytarabine (7 + 3). However, he subsequently developed biopsy-proven leukemia cutis without evidence of bone marrow relapse. On diagnosis, he was started on low-dose cytarabine and venetoclax. His past medical history was significant for *Pneumocystis jirovecii* pneumonia during induction chemotherapy, recurrent episodes of Gram-negative and Gram-positive bacteremia, dyslipidemia, and gastritis.

Computed tomography (CT) of the abdomen and pelvis was performed and demonstrated a mass-like thickening at the splenic flexure, raising concern for malignancy. Concurrent CT of the thorax revealed acute pulmonary emboli involving the right middle and lower lobar pulmonary arteries. Despite these findings, the patient denied abdominal pain, diarrhea, hematochezia, melena, fever, or chills.

One month later, colonoscopy demonstrated a friable 4 cm submucosal mass at the splenic flexure, and biopsies were obtained. Histopathologic examination revealed sheets of histiocytes which stained positive with periodic acid–Schiff stain. Basophilic cytoplasmic concretions highlighted by Von Kossa calcium and Perl’s iron stains were identified, consistent with Michaelis–Gutmann bodies. Ziehl–Neelsen staining for acid-fast bacilli was negative. The findings were diagnostic of colonic malakoplakia (Figure 1B).

The patient was subsequently evaluated by the Infectious Diseases service. Due to the absence of gastrointestinal symptoms and the limited evidence available to guide management of asymptomatic colonic malakoplakia, conservative management with close clinical monitoring was initially recommended. Potential treatment strategies discussed included antimicrobial agents with intracellular penetration, such as fluoroquinolones or trimethoprim-sulfamethoxazole, particularly if prolonged neutropenia necessitated antibacterial prophylaxis.

During hospitalization, the patient’s clinical course was complicated by relapsed AML confirmed on repeat bone marrow biopsy, persistent cytopenias, pulmonary embolism, recurrent febrile episodes, and progressive hypoxic respiratory failure with bilateral ground-glass pulmonary infiltrates concerning for atypical pneumonia or organizing pneumonia. Despite broad-spectrum antimicrobial therapy and supportive care, his respiratory status progressively deteriorated, ultimately requiring transfer to the intensive care unit. Although the patient denied gastrointestinal symptoms at presentation, we acknowledge that symptom attribution in patients with advanced hematologic malignancy is inherently difficult because systemic manifestations may overlap.

Following discussions regarding goals of care, the patient was elected for comfort-focused management and was transferred to the palliative care unit. He died approximately one month after the diagnosis of colonic malakoplakia.

### 3.3. Case Number Three: Renal Transplant Malakoplakia

A 30-year-old woman with a history of tuberous sclerosis and end-stage renal disease underwent a second deceased donor kidney transplant in 2021 following failure of a previous renal transplant performed ten years prior. Her maintenance immunosuppressive regimen consisted of prednisone, tacrolimus, and mycophenolate mofetil. Her additional medical history was significant for hypothyroidism, seizure disorder, polycystic ovarian syndrome, and chronic diarrhea attributed to mycophenolate therapy.

Several months after transplantation, she presented with a two-week history of headaches, malaise, nausea, vomiting, fevers, worsening diarrhea, abdominal pain, and reduced urine output. On admission, she was febrile and profoundly neutropenic with a leukocyte count below 0.5 × 10^9^/L. Blood and urine cultures both grew *Escherichia coli*. Renal ultrasonography demonstrated urothelial thickening involving the transplanted kidney, concerning for severe allograft pyelonephritis. Initial management included broad-spectrum antimicrobial therapy with meropenem in addition to supportive care.

Although tissue cultures were negative, repeated blood and urine cultures consistently isolated *Escherichia coli*, and the overall clinical, microbiological, radiological, and histopathological findings strongly supported chronic *E. coli*-associated renal allograft infection. Her hospitalization was prolonged and complicated by recurrent *E. coli* bacteremia and candidemia secondary to a peripherally inserted central catheter. She developed acute kidney injury requiring intermittent hemodialysis and hypoxemic respiratory failure necessitating intensive care unit admission, and significant uterine bleeding requiring dilation and curettage. Despite prolonged courses of antimicrobial therapy, she continued to experience relapsing bacteremia, raising concern for persistent infection involving the renal allograft with possible lobar nephronia or perinephric infection.

A renal biopsy demonstrated findings consistent with malakoplakia (Figure 1C). Tissue cultures for fungal and acid-fast organisms were negative. The overall clinicopathologic findings were considered highly suggestive of chronic allograft infection secondary to *Escherichia coli* in the setting of ongoing post-transplant immunosuppression.

In view of recurrent bacteremia, progressive graft dysfunction, persistent infection despite prolonged antimicrobial therapy, and dialysis dependence, the patient subsequently underwent transplant nephrectomy. Intraoperative findings demonstrated a markedly enlarged and swollen renal allograft with minimal residual viable renal parenchyma.

Following nephrectomy, the patient continued intravenous antimicrobial therapy with ceftriaxone and gradually improved clinically. She was discharged later that month on intermittent hemodialysis. At the time of discharge, the final diagnosis was chronic renal allograft infection with malakoplakia resulting in irreversible graft failure requiring transplant nephrectomy.

### 3.4. Case Number Four: UC Colon Malakoplakia

A 43-year-old man with a diagnosed case of ulcerative colitis was referred to the Infectious Diseases Clinic for evaluation of colonic malakoplakia identified on a recent sigmoidoscopy. His ulcerative colitis had been managed with long-term immunosuppressive therapy, including azathioprine and ustekinumab. Regular sigmoidoscopy demonstrated active ulcerative colitis with biopsy-proven malakoplakia (Figure 1D).

At presentation, the patient denied fever, chills, night sweats, cough, dyspnea, chest pain, or rash. History of tuberculosis exposure, recent travel, animal exposure, smoking, alcohol use, or recreational drug use was insignificant. Tuberculin skin testing and chest radiography were negative.

The patient also had a history of recurrent diarrheal episodes that had previously raised suspicion for possible *Clostridioides difficile* infection. However, repeated stool testing consistently demonstrated antigen and polymerase chain reaction positivity with toxin negativity, suggesting no active infection. Risk of developing recurrent *C. difficile* infection could be associated with prolonged fluoroquinolone therapy, so ciprofloxacin was avoided despite prior reports supporting its use in malakoplakia. The patient was started on rifaximin 200 mg orally twice daily.

At follow-up several months later, he reported worsening diarrhea while receiving rifaximin therapy, although treatment was continued. To improve tolerability, the dose was reduced to 200 mg once daily. Repeat stool testing for enteric pathogens and *Clostridioides difficile* was performed. Additional infectious work-up, including HIV, syphilis, and hepatitis viral serologies, was negative.

Repeat endoscopic biopsies obtained later that year demonstrated histologic resolution of malakoplakia following rifaximin therapy. Despite resolution of the malakoplakia, the patient continued to experience approximately five loose bowel movements daily, which were attributed to post-obstructive diarrhea in the setting of a colonic stricture related to longstanding ulcerative colitis.

Due to persistent symptoms and an advanced stage of ulcerative colitis, the patient subsequently underwent total colectomy. Surgical pathology demonstrated extensive ulcerative colitis without evidence of residual malakoplakia.

## 4. Discussion

The accumulation of defective macrophages carrying pathognomonic Michaelis–Gutmann bodies and undigested bacterial remains is the hallmark of malakoplakia, an uncommon chronic inflammatory illness. The disease’s etiology include immunological dysfunction, aberrant lysosome function in macrophages, and persistent bacterial infection affecting primarily immunocompromised people [11,12]. Defective lysosomal bactericidal function due to decreased cyclic guanosine monophosphate (cGMP) levels, abnormal immune regulation, and structural differences in bacterial organisms such as *Escherichia coli*, *Mycobacterium tuberculosis*, and *Proteus* spp. are among the suggested pathogenic mechanisms. The majority of cases occur in the genitourinary and gastrointestinal tracts [13,14,15,16].

We describe four distinct cases presenting with malakoplakia occurring in various immunocompromised conditions, such as liver transplantation, renal transplantation, acute myeloid leukemia, and ulcerative colitis. All of these patients were receiving biologic and immunosuppressive therapy of various types. Our findings are congruent with studies published by Seung Hyuk Yim et al. [17] and Michael Lee [4], indicating that malakoplakia can occur with a variety of different types of immunosuppression.

Despite the diversity of the underlying conditions, there were certain common clinicopathologic themes. Immunocompromised patients are more likely to develop malakoplakia. Immunosuppression is thought to impair macrophage bactericidal function by reducing intracellular cyclic guanosine monophosphate (cGMP) levels, resulting in defective phagolysosomal activity and incomplete degradation of phagocytosed bacteria [17,18]. It implies that the pathophysiology of malakoplakia may include compromised T lymphocyte activity. After stopping immunosuppressive treatment, recipients with malakoplakia have been observed to experience partial or total remission of phagocytic cell dysfunction [19].

The most effective diagnostic modality for malakoplakia is tissue biopsy. Histopathologic analysis shows Michaelis–Gutmann bodies, which are 2–10 mm lesions inside the cytoplasm of macrophages (known as von Hansemann cells). The Michaelis–Gutmann bodies stain positively for calcium and iron, and the macrophages express CD68 [20,21]. All our cases were confirmed by histopathological examination.

In contrast to existing literature, our study highlights a broad clinical spectrum of disease. Most previously published case series have focused on malakoplakia in a single immunocompromised state affecting the same organ in the entire series. Case series by Michael Lee et al. [4] present the pathologic diagnosis of malakoplakia in the gastrointestinal tract of 23 patients while Triozzi JL et al. [9] described renal transplant-associated malakoplakia worsened by infection. In contrast, our case series demonstrates a broader clinical spectrum involving multiple immunocompromised conditions and organ systems, including solid organ transplantation, hematologic malignancy, and autoimmune disease such as ulcerative colitis. Collectively, these cases underscore the heterogeneous clinical presentations of malakoplakia and emphasize the diagnostic challenges it poses in routine clinical practice.

In addition to diverse underlying conditions, our case series also demonstrates a wider range of clinical findings of malakoplakia. In our patient with AML, the disease mimicked a tumor-like mass on CT scan, while it was an incidental finding on routine sigmoidoscopy in the case of a patient suffering from ulcerative colitis. This highlights the variability of clinical manifestations of malakoplakia, making it a diagnostic challenge for clinicians.

A case series by Ali B. Abbasi et al. [22] reports two cases of malakoplakia after kidney transplant with *E. Coli* infections resolved after antimicrobial therapy. Conversely, our patient demonstrated a markedly different clinical outcome despite proper antimicrobial therapy and supportive care. Recurrent bacteremia, progressive graft dysfunction, and persistent infection despite prolonged antimicrobial therapy led to transplant nephrectomy. This observation highlights that similar underlying clinical conditions associated with malakoplakia may result in markedly different clinical outcomes despite comparable therapeutic approaches. Such variability underscores the challenges inherent in the management of malakoplakia and emphasizes the need for individualized treatment strategies based on the patient’s clinical presentation, disease severity, and underlying comorbidities.

Reduced immunosuppressive treatments, antibiotics, and, in rare cases, surgery are used to treat malakoplakia. Because TMP/SMX and fluoroquinolones can buildinside macrophages, they are the recommended antibiotics for treating malakoplakia. In addition to antibiotics, bethanechol chloride may be used as an adjunctive treatment because it has been demonstrated to raise cGMP levels. Because the pathogenesis of malakoplakia involves defective intracellular bacterial killing by macrophages, antimicrobial agents with good intracellular penetration, particularly fluoroquinolones and trimethoprim-sulfamethoxazole, are generally preferred. Selection of therapy should also consider microbiological culture results, antimicrobial susceptibility, patient comorbidities, drug interactions, and tolerance. In our series, treatment decisions were individualized according to these clinical factors [23]. Treatment response determined the treatment duration in our cases. In more advanced cases, treatment with antibiotics alone is usually not sufficient, and surgery may be necessary [24,25]. In one case, failure of antimicrobial therapy to achieve adequate disease control necessitated nephrectomy.

With the advent of recent technologies in xAI, gastrointestinal lesions can be detected and localized in endoscopic imaging. Being rare and similar to other gastrointestinal diseases, histopathological examination is necessary to confirm a diagnosis of malakoplakia, although these technologies have proven to have a good diagnostic performance in various inflammatory/neoplastic diseases. However, AI can be used during endoscopy to help detect suspicious lesions that need to be biopsied earlier.

Since immunosuppressed patients are predisposed to malakoplakia, pathologists should be aware of this and maintain an elevated index of suspicion for the diagnosis.

There are some limitations with the present study. The results of this case series are not generalizable to all patients, as it is a small, retrospective case series. In addition, there were no specific guidelines for the treatment of malakoplakia and management was individualized based on clinical context. This study is also limited by the fact that the available literature consists exclusively of case reports, which are inherently at high risk of bias due to selective reporting. Moreover, several reports do not include core data on risk factors and outcomes. Our cases add to the evidence towards diversity of clinical presentations, histopathology, and prognosis of malakoplakia in immunocompromised hosts.

## 5. Conclusions

Malakoplakia is an uncommon chronic inflammatory condition that can affect multiple organ systems. It occurs most often in patients with chronic illnesses or immunosuppressive conditions. Although it is a benign and often self-limited condition, presentation as a mass lesion may raise suspicion of malignancy. Antibiotics form the mainstay of treatment. We demonstrate that it can manifest in many different clinical contexts, including solid organ transplantation, hematologic malignancy, and chronic inflammatory disorders under immunosuppressive therapy. For optimal outcomes, early recognition and histopathologic confirmation are essential, and infectious and neoplastic mimics should be excluded.

## Figures and Tables

**Table 1:** Summary of malakoplakia cases reported in the literature.

Domain	Variable	Category/Details	*n* (%)
Demographics	Total cases	—	52 (100%)
	Mean age	—	50.0 years
	Sex	Male	26 (50.0%)
		Female	26 (50.0%)
Anatomic site	GI tract	Colon, rectum, stomach, intestine	40 (76.9%)
	GU tract	Kidney, bladder, prostate	10 (19.2%)
	Head and neck	Maxilla	1 (1.9%)
	Skin	Cutaneous	1 (1.9%)
Underlying condition	Malignancy	Solid tumors, hematologic cancers	17 (32.7%)
	Transplantation	Renal/liver/other SOT	14 (26.9%)
	Autoimmune disease	IBD, SLE, etc.	11 (21.2%)
	Drug-induced immunosuppression	Steroids, biologics, immunomodulators	9 (17.3%)
	Diabetes mellitus	—	7 (13.5%)
	Active infection-related state	Chronic/acute infections	4 (7.7%)
	HIV infection	—	1 (1.9%)
Immunosuppression status	On active immunosuppressive therapy	Tacrolimus, MMF, steroids, ATG, biologics	16 (30.8%)
Microbiology	Culture-positive cases	—	9 (17.3%)
	Common organisms	*E. coli*, *Klebsiella pneumoniae*, *Enterococcus faecium*, *VRE*, *Providencia* spp., *Candida albicans*	—
Treatment	Antimicrobial therapy	Antibiotics used	20 (38.5%)
		Ciprofloxacin, TMP-SMX, cefepime, ceftriaxone, doxycycline, minocycline, tetracycline, levofloxacin, rifaximin, cefpodoxime, daptomycin	—
	Surgical management	Resection/nephrectomy/colectomy	10 (19.2%)
Outcome	Clinical improvement/resolution	—	24 (46.2%)
	Mortality	—	1 (1.9%)
	Not reported/unknown	—	27 (51.9%)

“Patients may have had more than one underlying immunocompromising condition; therefore, percentages do not total 100%.”

**Table 2:** Clinical Characteristics, Management, and Outcomes of Four Patients with Malakoplakia.

Case	Age/Sex	Site	Underlying Disease	Immunosuppressive/Immunomodulatory Therapy	Clinical Presentation	Histopathology	Treatment	Outcome
1	56/M	Rectum	Liver transplant for primary biliary cholangitis	Tacrolimus, mycophenolate mofetil	Upper GI bleeding; rectal malakoplakia	Michaelis–Gutmann bodies	TMP-SMX for 3 months	Complete clinical and histologic resolution
2	70/M	Splenic flexure (colon)	Relapsed AML with leukemia cutis	Low-dose cytarabine, venetoclax	Asymptomatic GI presentation	Michaelis–Gutmann bodies	Observation	Death from progressive AML
3	30/F	Renal allograft	Kidney transplant recipient; tuberous sclerosis, ESRD	Prednisone, tacrolimus, mycophenolate mofetil	Sepsis and recurrent *E. coli* bacteremia	Renal malakoplakia	Meropenem, prolonged ceftriaxone therapy, and transplant nephrectomy	Resolution of infection following transplant nephrectomy; permanent loss of renal allograft requiring long-term hemodialysis.
4	43/M	Colon	Ulcerative colitis	Azathioprine, ustekinumab	Chronic diarrhea	Colonic malakoplakia	Rifaximin	Histologic resolution; colectomy for advanced ulcerative colitis

## Data Availability

The original contributions presented in this study are included in the article. Further inquiries can be directed to the corresponding author(s).
